# Pediatric high-grade glioma *MYCN* is frequently associated with Li-Fraumeni syndrome

**DOI:** 10.1186/s40478-022-01490-w

**Published:** 2023-01-06

**Authors:** Léa Guerrini-Rousseau, Arnault Tauziède-Espariat, David Castel, Etienne Rouleau, Philipp Sievers, Raphaël Saffroy, Kévin Beccaria, Thomas Blauwblomme, Lauren Hasty, Franck Bourdeaut, Jacques Grill, Pascale Varlet, Marie-Anne Debily

**Affiliations:** 1 Department of Child and Adolescents Oncology, Gustave Roussy, Université Paris-Saclay, 94805 Villejuif, France; 2 Molecular Predictors and New Targets in Oncology, INSERM U981, Gustave Roussy, Université Paris-Saclay, 94805 Villejuif, France; 3 Department of Neuropathology, GHU Paris-Psychiatrie et Neurosciences, Sainte-Anne Hospital, 1, rue Cabanis, 75014 Paris, France; 4 Inserm, UMR 1266, IMA-Brain, Institut de Psychiatrie et Neurosciences de Paris, Paris, France; 5 Department of Biology and Pathology, Tumor Genetics Service, Gustave Roussy, Université Paris-Saclay, 94805 Villejuif, France; 6 Department of Neuropathology, Institute of Pathology, University Hospital Heidelberg, Heidelberg, Germany; 7 Clinical Cooperation Unit Neuropathology, German Consortium for Translational Cancer Research (DKTK), German Cancer Research Center (DKFZ), Heidelberg, Germany; 8 Department of Biochemistry and Oncogenetic, Paul Brousse Hospital, 94804 Villejuif, France; 9 Department of Pediatric Neurosurgery, Necker Hospital, APHP, Université Paris Cité, 75015 Paris, France; 10 INSERM U830, Laboratory of Translational Research in Pediatric Oncology, SIREDO Pediatric Oncology Center, Curie Institute, Paris, France; 11 Univ. Evry, Université Paris-Saclay, 91000 Evry, France

In the current World Health Organization (WHO) Classification of Tumors of the Central Nervous System (CNS), pediatric high-grade gliomas (HGGs), *IDH-* and histone H3*-*wildtype (WT) are divided into three molecular subtypes: RTK1, RTK2 and MYCN [2]. HGG-MYCN present recurrent histopathological characteristics (nodular pattern with embryonal cells), genetic features (frequent *MYCN* and/or *ID2* amplification, and somatic *TP53* mutations) [1, 5, 6], and a specific DNA-methylation profile. Li-Fraumeni syndrome (LFS) encompasses a wide variety of primary brain tumors. They include HGGs, *IDH-*WT with *MYCN* amplification, but only one of them was reported in the literature as a HGG-MYCN by DNA-methylation profiling [3, 4]. The proportion of specimens from the epigenetic subgroup HGG-MYCN associated with LFS remains an unanswered question. The aim of this study was to analyze the somatic and germline status of *TP53* and the DNA-methylation profile (using the v12.5 of the Heidelberg Brain Tumor classifier) from a series of HGG-MYCN. From a series of 151 pediatric HGGs, we selected 11 cases suspected of belonging to a *MYCN* subtype based on histopathology and *MYCN* amplification (identified by FISH analysis, *cf.* Additional file 1: Fig. S1).

The clinical, genetic and epigenetic characteristics of the cohort are summarized in Table 1. Of the 11 HGGs initially diagnosed as HGG-MYCN, each one presented a p53 overexpression using immunohistochemistry and harbored a somatic *TP53* pathogenic variant (PV) (*cf.* Additional file 2: Methods). The tumor classification based on DNA-methylation confirmed the diagnosis of the pediatric HGG-MYCN subtype in 5/7 cases with high calibrated scores (> 0.9) having sufficient DNA available for the analysis. The two remaining cases were classified as pediatric HGG, not otherwise specified, subtype A (despite the presence of a *MYCN* amplification detected by FISH and copy number variation of whole exome sequencing data) and HGG-MYCN with a low calibrated score (0.20). However, using t-distributed stochastic neighbor embedding (t-SNE) analysis, all cases clustered within or in close vicinity to HGG-MYCN (Fig. 1).

**Table 1:** Clinical, genetic and epigenetic details of the cohort

Reference case	Age at diagnosis (y), sex	Tumor location	Somatic *TP53* pathogenic variation (NM_000546.5)	Methylation-based classification (calibrated score) v12.5	Germline *TP53* status	Status at the end of follow-up, OS (y)
1	4.3, F	Pons	c.469G>T; p.(Val157Phe), Exon 5	Diffuse paediatric-type HGG, MYCN subtype (0.99)	Mutated: c.469G>T; p.(Val157Phe), Exon 5	Dead (0.4)
2	5.2, M	Multifocal (cerebellum. mesencephalic. bulbar and thalamic)	c.742C>G; p.(Arg248Gly), Exon 07	Diffuse paediatric-type HGG, MYCN subtype (0.90)	Mutated: c.742C>G; p.(Arg248Gly), Exon 07	Dead (1)
3	3.4, M	Left fronto-parietal lobe	c.701A>G; p.(Tyr234Cys), Exon 07	Diffuse paediatric-type HGG, MYCN subtype (0.99)	Mutated: c.701A>G; p.(Tyr234Cys), Exon 07	Dead (1.7)
4	3.3, M	Left frontal lobe	c.731G>A; p.(Gly244Asp), Exon 07	Diffuse paediatric-type HGG, MYCN subtype (0.99)	Mutated; c.731G>A, p.(Gly244Asp)	Dead (1.5)
5	4.5, M	Left thalamus	c. 743G>A; p.(Arg248Gln), Exon 07	Diffuse paediatric-type HGG, MYCN subtype (0.99)	WT	Dead (0.3)
6	7.6, F	Pons	c.817C>T; p.(Arg273Cys), Exon 8	Diffuse paediatric-type HGG, MYCN subtype (0.20)	WT	Dead (0)
7	3.2, F	Right thalamus	c.853G>A; p.(Glu285Lys), Exon 08	Diffuse paediatric-type HGG, H3 wildtype and IDH WT, Subtype A (0.99)	WT	Dead (0.7)
8	3.1, M	Pons	c.916C>T; (p.Arg306Ter), Exon 8 c.632C>T; p.(Thr211Ile), Exon 6	NA	WT	Dead (0.5)
9	1.3, F	Pons	c.524G>A; p.(Arg175His), Exon 08	NA	WT	Dead (0.2)
10	2.8, F	Pons	c.742C>T; p.(Arg248Trp), Exon 7	NA	WT	Dead (NA)
11	4.4, M	Pons	c.844C>A; p.(Arg282Trp), Exon 8	NA	WT	Dead (0.7)

*F* female, *HGG* high-grade glioma, *M* male, *NA* not available, *OS* overall survival, *WT* wildtype, *y* years-old

**Fig. 1:**
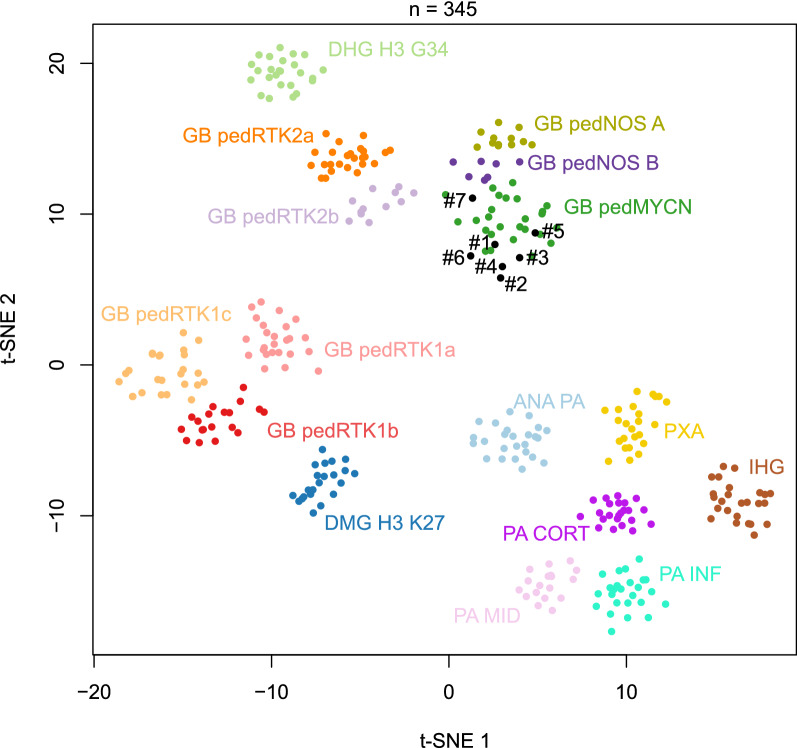
t-Distributed stochastic neighbor embedding (t-SNE) analysis of DNA methylation profiles of the investigated tumors alongside selected reference samples. Reference DNA methylation classes: high-grade astrocytoma with piloid features (ANA PA); diffuse high-grade glioma, H3.3 G34 mutant (DHG H3 G34); diffuse midline glioma H3 K27M mutant (DMG H3 K27); pediatric glioblastoma, IDH wildtype, subclass MYCN (GB pedMYCN); pediatric glioblastoma, IDH wildtype, subclass not otherwise specified sutbype A (GB pedNOS A); pediatric glioblastoma, IDH wildtype, subclass not otherwise specified sutbype B (GB pedNOS B); pediatric glioblastoma, IDH wildtype, subclass RTK1a (GB pedRTK1a); pediatric glioblastoma, IDH wildtype, subclass RTK1b (GB pedRTK1b); pediatric glioblastoma, IDH wildtype, subclass RTK1c (GB pedRTK1c); pediatric glioblastoma, IDH wildtype, subclass RTK2a (GB pedRTK2a); pediatric glioblastoma, IDH wildtype, subclass RTK2b (GB pedRTK2b); infant-type hemispheric glioma (IHG); hemispheric pilocytic astrocytoma (PA CORT); infratentorial pilocytic astrocytoma (PA INF); midline pilocytic astrocytoma (PA MID); pleomorphic xanthoastrocytoma (PXA)

Four out of seven (57%) patients had a *TP53* germline PV. All four of these four cases presented a high calibrated score for the HGG-MYCN methylation class. The information concerning a family history, or for a predisposition to cancer was not available for all patients. Among the families explored in genetic counseling, one patient presented a de novo PV (case #4) and genetic analyses are currently in progress for parents of another patient (case #2) having a family history of malignant sarcoma in the grandfather. No *TP53* germline mutation was observed in the other cases.

Previously, only one case report of HGG-MYCN in LFS has been reported in the literature [3]. Based on the high prevalence of somatic *TP53* PV in epigenetically confirmed HGG-MYCN (100% of cases in our series, 67% in the study which initially described the methylation class [1]), we demonstrate for the first time that this tumor type is frequently associated with LFS and may constitute the mode of revelation for this genetic predisposition syndrome. LFS cases do not seem to form a distinct subcluster in the DNA-methylation based classification of CNS tumors compared to those without a *TP53* germline mutation. Whereas *MYCN* amplification and *TP53* PV are enriched in HGG-MYCN, these alterations are not specific to this subgroup and may be encountered within other subtypes of pediatric HGGs (RTK1, RTK2) [1]. In this current study, one case was classified as a pediatric HGG, not otherwise specified, subtype A (with a high calibrated score) but clustered in close vicinity to HGG-MYCN using t-SNE analysis. This result highlights the fact that the epigenetic boundaries between all subtypes (eight different methylation classes defined in the v12.5 of the DKFZ classifier) of pediatric HGGs are still in progress, and potentially argues that the three initial subgroups defined by Korshunov et al*.* (and particularly cases included in the HGG-MYCN subgroup which do not present *MYCN* amplification and *TP53* mutation) are probably redefined in other methylation classes.

To conclude, a constitutional analysis of *TP53* and a genetic counseling should be proposed to all patients with proven HGG-MYCN harboring a *TP53* somatic alteration. Additional cases are needed to determine if the HGG-MYCN associated with LFS forms a distinct methylation subclass from those without a germline mutation of *TP53,* as was described for Primary mismatch repair deficient *IDH*-mutant astrocytoma in the v12.3 of the DKFZ classifier. Moreover, further studies are needed to determine if clinical (pediatric tumor), histopathological (HGG with embryonal features), and genetic (*TP53* PV and *MYCN* amplification) features may constitute alternative diagnostic criteria by DNA-methylation profiling for a diagnosis of HGG-MYCN.

## Supplementary Information

**Additional file 1. Fig. S1**: Histopathological features. Black scale bars represent 1 mm (A), 100 μm (B) and 50 μm (C to K). (A-C-E-G-H-I-K-M) Diffuse and solid proliferation with several nodules infiltrating the brain parenchyma. Undifferenciated proliferation composed of hyperchromatic cells presenting anisocaryotic nuclei with numerous apoptotic bodies (HPS, x400 magnification). (B-D-F-H-J-L-N) Nuclear accumulation of p53 (x400 magnification). Black scale bars represent 50 μm.

**Additional file 2.** Methods used in this series.
