# Supplementary figures and images for: Pediatric high-grade glioma MYCN is frequently associated with Li-Fraumeni syndrome
Source: Acta Neuropathol Commun. 2023 Jan 6;11:3. doi: 10.1186/s40478-022-01490-w (PMC9817308; doi:10.1186/s40478-022-01490-w)

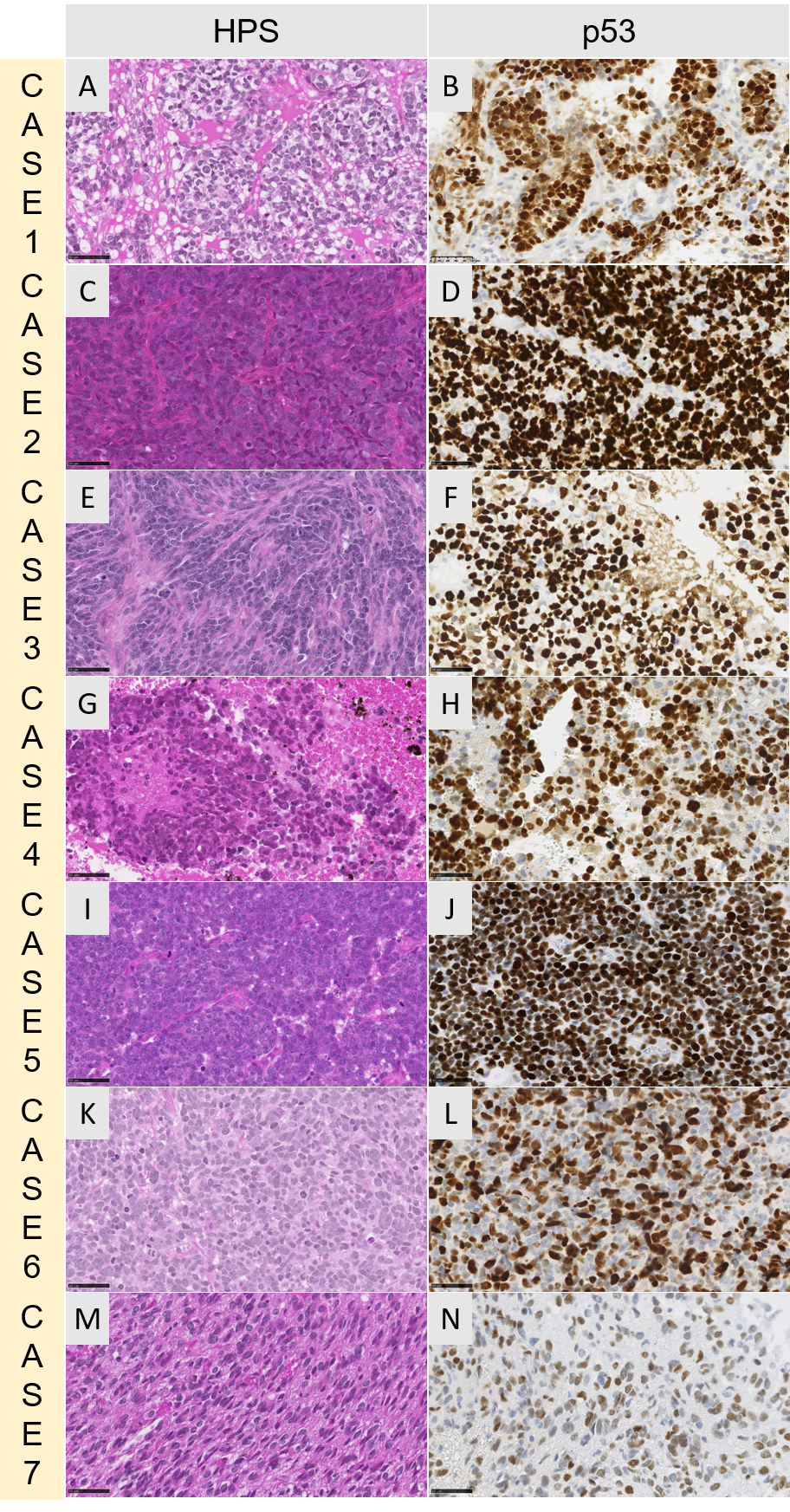

Supplement: Supplementary file 1 — Additional file 1. Fig. S1: Histopathological features. Black scale bars represent 1 mm (A), 100 μm (B) and 50 μm (C to K). (A-C-E-G-H-I-K-M) Diffuse and solid proliferation with several nodules infiltrating the brain parenchyma. Undifferenciated proliferation composed of hyperchromatic cells presenting anisocaryotic nuclei with numerous apoptotic bodies (HPS, x400 magnification). (B-D-F-H-J-L-N) Nuclear accumulation of p53 (x400 magnification). Black scale bars represent 50 μm. [file 40478_2022_1490_MOESM1_ESM.tif]
