# Supplementary material for: Pediatric high-grade glioma MYCN is frequently associated with Li-Fraumeni syndrome
Source: Acta Neuropathol Commun. 2023 Jan 6;11:3. doi: 10.1186/s40478-022-01490-w (PMC9817308; doi:10.1186/s40478-022-01490-w)
Supplement: Supplementary file 2 — Additional file 2. Methods used in this series. [file 40478_2022_1490_MOESM2_ESM.docx]

**Methods**

**Central histopathological review and immunohistochemistry**

The central pathology review was performed conjointly by two neuropathologists (ATE and PV). A representative paraffin block was selected for each case. Unstained 3-μm-thick slides of formalin-fixed, paraffin-embedded tissues were obtained and submitted for immunostaining. The following primary antibody was used: p53 (1:5000, clone DO-1, Clinisciences, Nanterre, France). External positive and negative controls were used for all antibodies.

**Fluorescent *In Situ* Hybridization (FISH) and Molecular Analyses**

FISH analyses for *MYCN* gene amplification using the ZytoLight SPEC *MYCN* probe (Zytovision, Bremerhaven, Germany) were carried out in all cases. Fluorescent signals were counted in 100 tumoral nuclei with the DM600 Leica fluorescent microscope (Leica Biosystems, Richmond, IL).

**Detection of somatic *TP53* mutation by Next-Generation Sequencing**

Genomic DNA was extracted from formalin-fixed and paraffin-embedded (FFPE) tissue or from blood samples. For the TP53 variant confirmation either custom panel on SureSelect QXT HS kit (Agilent Technologies, Santa Clara, CA United States) for ADN extracted from blood samples or a custom panel on SureSelect XT HS kit (Agilent Technologies, Santa Clara, CA United States) from FFPE tissue samples. The QXT HS panel covered 42 genes and the XT HS panel covered 35 genes. The full preparation was done on a Bravo equipment option B (Agilent) for QXT HS kit and Magnis (Agilent) for XT HS kit. Pooled libraries containing captured DNA fragments were subsequently sequenced on an NextSeq platform (Illumina) as 2 × 150-bp paired-end reads. Sequences were demultiplexed using an in-house tool. The data analysis pipeline included the following algorithms developed internally: BWA-MEM v-0.7.12 for read alignment to the hg19 human reference genome and Samtools v-1.2 and Picard-tools v-1.139 for PCR duplicate quantification and removal. GATK Haplotype v-3.4-46, snpEff v-4.0 and MutaCaller-1.7 (home pileup internally developed) were used for variant calling and classification. Variants were called with a minimum allelic frequency threshold of 1% for already classified variants (those known in the internal database) and 5% for non-classified variants, and a read depth threshold of 30X for the total reads at the variant location and at least 10X for the variant.

Several filters were applied to further select for potential relevant variants among the called variants. The population databases Exac and gnomAd were used to automatically filter out polymorphism as soon as the population frequency was higher than 0.5%. Non-classified variants (not known in the internal database) were excluded if the intrarun recurrency was superior to 4.
